# Supplementary material for: A high-throughput, 28-day, microfluidic model of gingival tissue inflammation and recovery
Source: Commun Biol. 2023 Jan 23;6:92. doi: 10.1038/s42003-023-04434-9 (PMC9870913; doi:10.1038/s42003-023-04434-9)
Supplement: Supplementary file 2 — Supplementary Information [file 42003_2023_4434_MOESM2_ESM.pdf]

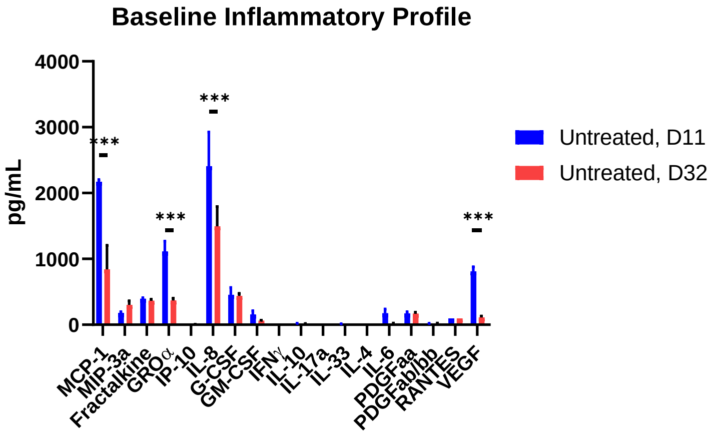

*Supplementary Figure 1. An inflammatory biomarker panel shows a profile of secreted cytokines in media collected from the bottom channel on day 11, the first day of plateau, and day 32, the last day of the culture. Almost all biomarker levels decreased over the course of the experiment, with none significantly increasing over the same time period. N=4 for D11, N=3 for D32. *** = P<0.0001 .*

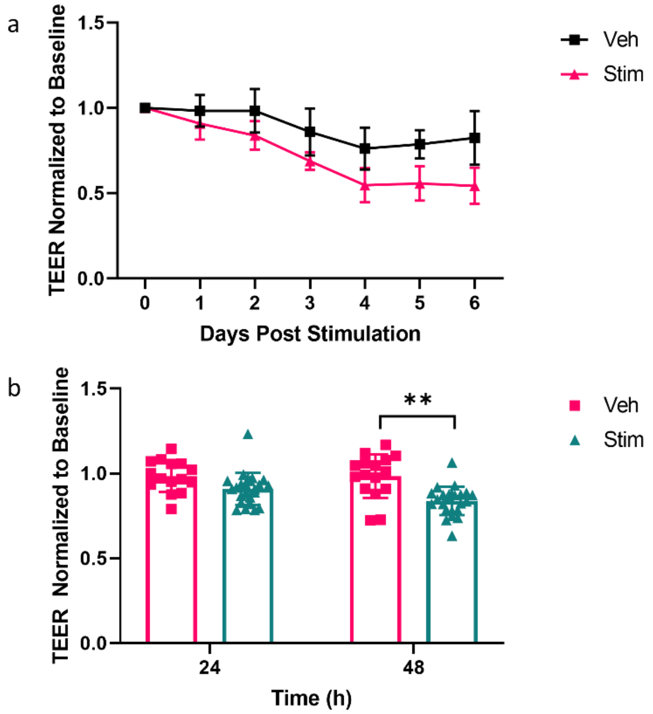

Supplementary Figure 2. ***a.*** *By 6 days post-stimulation, Stimulated (Stim) devices lost 45% of barrier function and were statistically significant from untreated and vehicle (Veh. BSA) conditions (p<0.0001). Devices were stimulated around Day 10.* ***b.*** *A decrease in TEER was seen as early as 24 hours, when a drop in barrier function was detected relative to untreated control devices (p<0.05). Data are averaged across 3 experiments (N=3-12 per experiment). ** = p <0.002.*

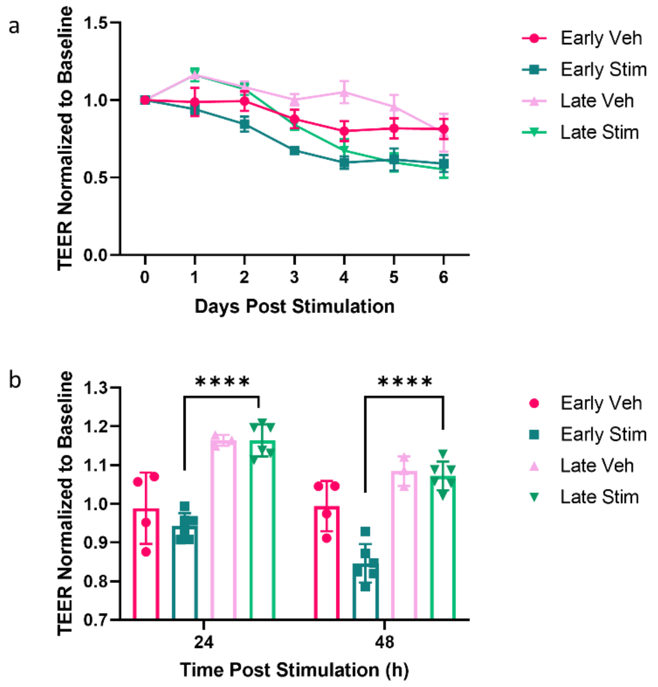

*Supplementary Figure 3. **a.** Barrier function response to stimulation was more immediate in devices stimulated at day 10 (Early Stim) compared to devices that were stimulated after 21 days in culture (Late Stim), p<0.0001. By 4 days post treatment, the normalized TEER values of Early Stim and Late Stim converged, and by 6 days post stimulation, Early Stim and Late Stim devices lost 41% and 45% of their barrier function, respectively, p<0.0001 compared to controls. **b.** Interestingly, Late Veh. and Late Stim both increased in TEER during the first 48 hours post treatment compared to Early stim, which lost 15% of barrier function compared to vehicle within 48 hours. ***= p <0.0001.*

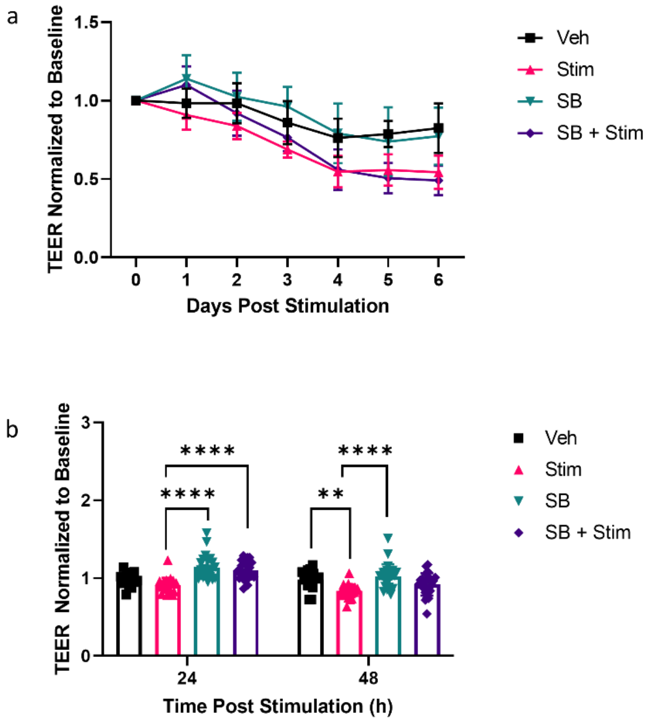

Supplementary Figure 4. ***a.*** *For 24 hours, SB + Stim devices maintained barrier function compared to Stim conditions (p < 0.0001). By 3 days post-stimulation SB TEER was higher than TEER of SB + Stim devices (p < 0.01). By 6 days post-stimulation, SB + Stim devices lost a similar amount of barrier function compared to Stim devices, which ultimately lost 46% of their barrier function compared to controls (p<0.0001).* ***b.*** *Barrier function improves for 24 hours after devices were exposed to small molecule inhibitor SB203580 (SB) relative to controls, but does not have a protective effect of Stim conditions by 48 hours. Data presented is averaged across 3 experiments (N=3-6 per experiment). ** = p<0.005, *** = p <0.0001.*

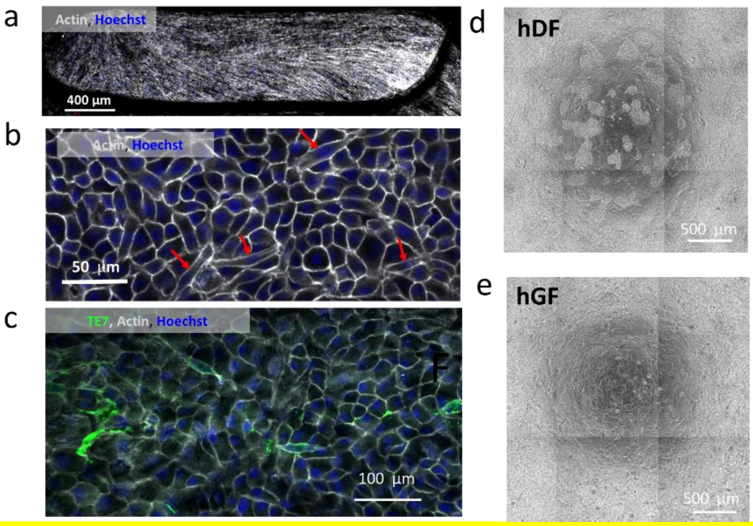

Supplementary Figure 5 Characterization of fibroblasts in the MOUTH model. a) hGFs formed a confluent monolayer prior to introducing the hOK. After 3-4 weeks of triculture conditions, the hGF were often difficult to distinguish from the other two tissue types but could be seen b) sporadically throughout the basal layers due to their elongated morphology and c) TE-7 expression. When co-cultured with hOK in Transwells for seven days in custom media, d)human dermal fibroblasts (hDF) resulted in pronounced piling and surface topography compared to hOKs co-cultured with e) hGFs.

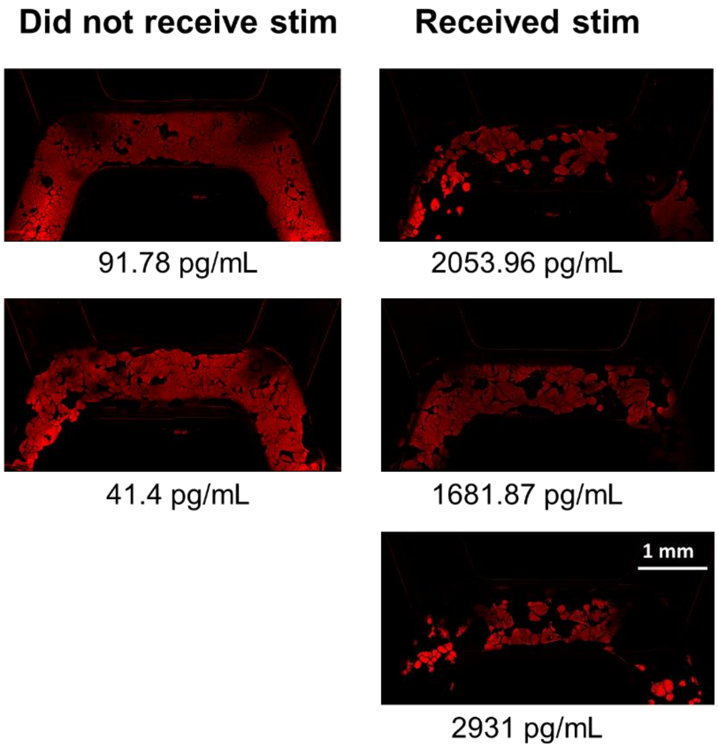

Supplementary Figure 6. Representative images of hMVECs in devices that did and did not receive stimulation indicate that the number of hMVEC can vary across a single condition and that the PGE-2 values are likely more correlated to cell viability than cell number. 1 mm scale bar is representative for all figure panels.

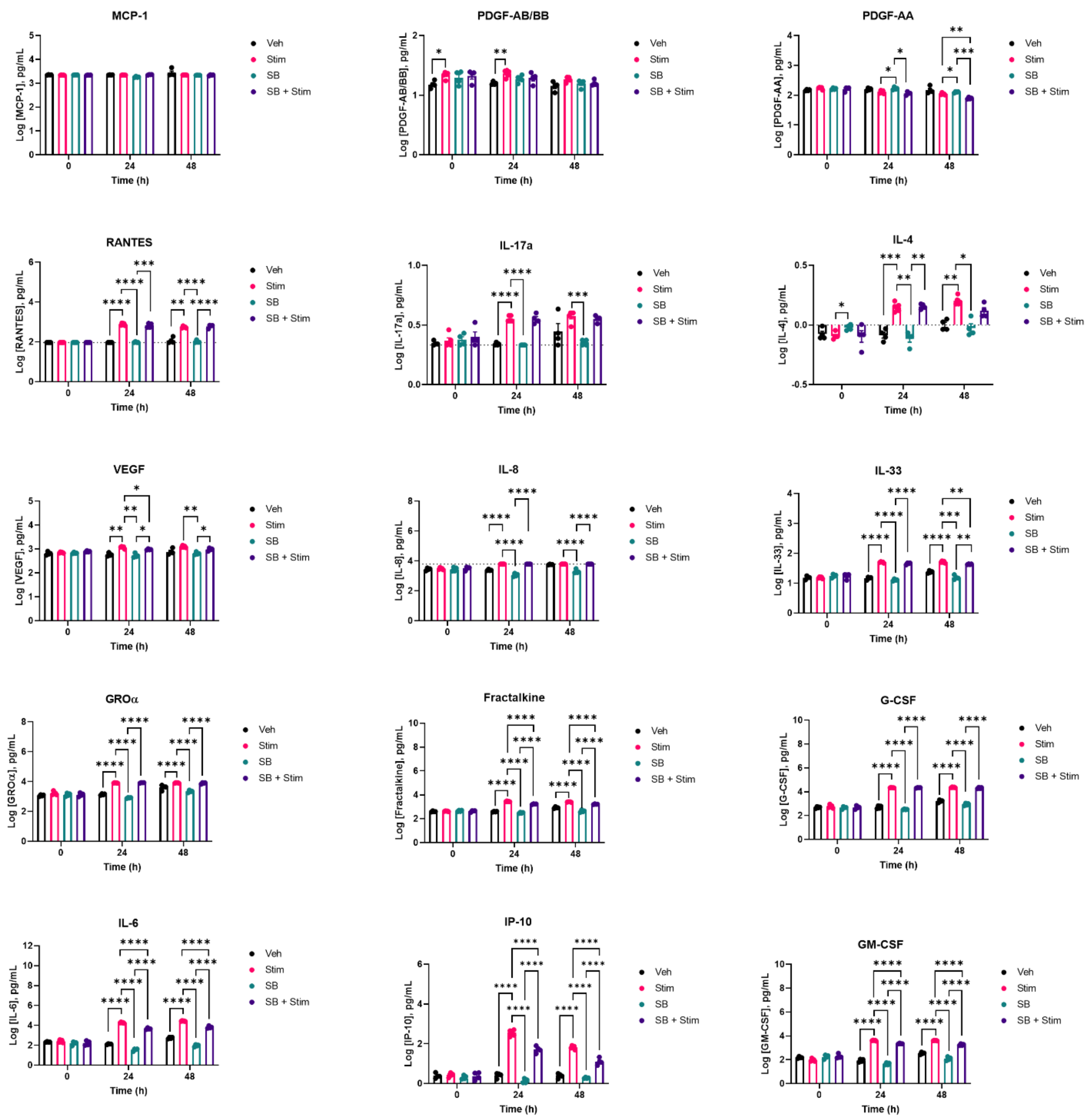

Supplementary Figure 7 *Inflammatory stimulation had a measurable impact on cytokine secretions in the MOUTH model. Fractalkine, GM-CSF, G-CSF, RANTES, VEGF, IL-33, IP-10, and IL-6 were all upregulated following inflammatory stimulation, a result that was inhibited when pre-treated with SB by within at least 48 hours. GROα, IL-8, and IL-4 were also upregulated following inflammation stimulation, but not protected when pre-treated with SB. MCP-1, G-CSF, PDGFaa, and PDGFab/bb expression levels were not significantly affected by IL-1β/TNFα1B/TNFα stimulation.*

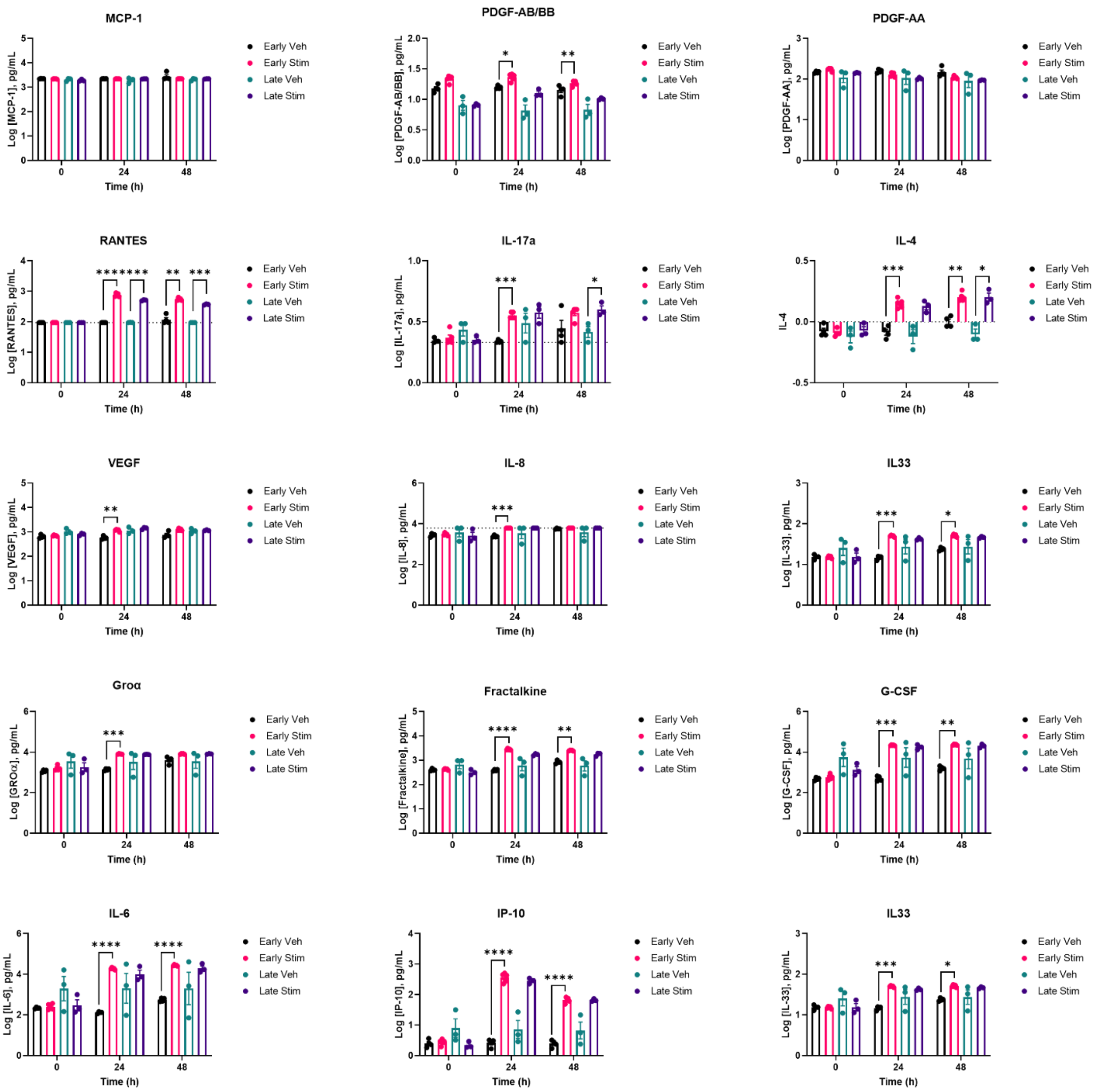

Supplementary Figure 8 Early inflammatory stimulation had a measurable impact on cytokine and chemokine secretions with late stimulation trending similarly over several cytokine and chemokine expression levels.

Supplementary Video 1. A z-stack confocal video of MOUTH tissue cultured for approximately 30 days shows the full tissue thickness from the microvascular endothelial cells on the bottom side of the membrane through the multi-layered gingival keratinocytes. The full stack is 124 µm thick with the keratinocytes comprising about 88 µm of the total. Tissue was labeled for actin cytoskeleton (gray), DNA (blue), and von Willebrand Factor (red). Scale bar = 100 µm.
