# Supplementary material for: A high-throughput, 28-day, microfluidic model of gingival tissue inflammation and recovery
Source: Commun Biol. 2023 Jan 23;6:92. doi: 10.1038/s42003-023-04434-9 (PMC9870913; doi:10.1038/s42003-023-04434-9)
Supplement: Supplementary file 3 — Description of Additional Supplementary Files [file 42003_2023_4434_MOESM3_ESM.pdf]

Description of Additional Supplementary Files

File name: Supplementary Data 1

Description: The source data behind the graphs in the paper, including Fig 1D, Fig 2B-e, Fig 3B-E, Fig 4B-E, Fig 5B-C, SFig 1, SFig 2A-B, SFig 3B, SFig 4B, SFig 7, and SFig 8. Each figure dataset is in it's own tab.

File name: Supplementary Video 1

Description: A z-stack confocal video of MOUTH tissue cultured for approximately 30 days shows the full tissue thickness from the microvascular endothelial cells on the bottom side of the membrane through the multi-layered gingival keratinocytes. The full stack is 124 µm thick with the keratinocytes comprising about 88 µm of the total. Tissue was labeled for actin cytoskeleton (gray), DNA (blue), and von Willebrand Factor (red). Scale bar = 100 µm.
