# Supplementary material for: A high-throughput, 28-day, microfluidic model of gingival tissue inflammation and recovery
Source: Commun Biol. 2023 Jan 23;6:92. doi: 10.1038/s42003-023-04434-9 (PMC9870913; doi:10.1038/s42003-023-04434-9)
Supplement: Supplementary file 6 — Reporting Summary [file 42003_2023_4434_MOESM6_ESM.pdf]

nature portfolio

Corresponding author(s): Else M. Vedula

Last updated by author(s): Dec 2, 2022

Reporting Summary

Nature Portfolio wishes to improve the reproducibility of the work that we publish. This form provides structure for consistency and transparency in reporting. For further information on Nature Portfolio policies, see our Editorial Policies and the Editorial Policy Checklist.

Statistics

For all statistical analyses, confirm that the following items are present in the figure legend, table legend, main text, or Methods section.

n/a	Confirmed	
☐	☒	The exact sample size (*n*) for each experimental group/condition, given as a discrete number and unit of measurement
☐	☒	A statement on whether measurements were taken from distinct samples or whether the same sample was measured repeatedly
☐	☒	The statistical test(s) used AND whether they are one- or two-sided<br>*Only common tests should be described solely by name; describe more complex techniques in the Methods section.*
☒	☐	A description of all covariates tested
☐	☒	A description of any assumptions or corrections, such as tests of normality and adjustment for multiple comparisons
☐	☒	A full description of the statistical parameters including central tendency (e.g. means) or other basic estimates (e.g. regression coefficient) AND variation (e.g. standard deviation) or associated estimates of uncertainty (e.g. confidence intervals)
☐	☒	For null hypothesis testing, the test statistic (e.g. *F*, *t*, *r*) with confidence intervals, effect sizes, degrees of freedom and *P* value noted<br>*Give P values as exact values whenever suitable.*
☒	☐	For Bayesian analysis, information on the choice of priors and Markov chain Monte Carlo settings
☒	☐	For hierarchical and complex designs, identification of the appropriate level for tests and full reporting of outcomes
☒	☐	Estimates of effect sizes (e.g. Cohen's *d*, Pearson's *r*), indicating how they were calculated

Our web collection on statistics for biologists contains articles on many of the points above.

Software and code

Policy information about availability of computer code

Data collection	xPONENT software was used to acquire Luminex data.
Data analysis	Commercially available GraphPad PRISM software, version 9.0.2, was used for statistical analysis

For manuscripts utilizing custom algorithms or software that are central to the research but not yet described in published literature, software must be made available to editors and reviewers. We strongly encourage code deposition in a community repository (e.g. GitHub). See the Nature Portfolio guidelines for submitting code & software for further information.

Data

Policy information about availability of data

All manuscripts must include a data availability statement. This statement should provide the following information, where applicable:

- Accession codes, unique identifiers, or web links for publicly available datasets
- A description of any restrictions on data availability
- For clinical datasets or third party data, please ensure that the statement adheres to our policy

Source data for Figs 2b-e, 3b-3, 4b-e, and 5b-c, as well as Supplementary Figs 1-4, 7 and 8 are included in supplementary information files. All other data supporting the findings of this study are available from the corresponding author on request.

Human research participants

Policy information about studies involving human research participants and Sex and Gender in Research.

Reporting on sex and gender	*Use the terms sex (biological attribute) and gender (shaped by social and cultural circumstances) carefully in order to avoid confusing both terms. Indicate if findings apply to only one sex or gender; describe whether sex and gender were considered in study design whether sex and/or gender was determined based on self-reporting or assigned and methods used. Provide in the source data disaggregated sex and gender data where this information has been collected, and consent has been obtained for sharing of individual-level data; provide overall numbers in this Reporting Summary. Please state if this information has not been collected. Report sex- and gender-based analyses where performed, justify reasons for lack of sex- and gender-based analysis.*
Population characteristics	*Describe the covariate-relevant population characteristics of the human research participants (e.g. age, genotypic information, past and current diagnosis and treatment categories). If you filled out the behavioural & social sciences study design questions and have nothing to add here, write "See above."*
Recruitment	*Describe how participants were recruited. Outline any potential self-selection bias or other biases that may be present and how these are likely to impact results.*
Ethics oversight	*Identify the organization(s) that approved the study protocol.*

Note that full information on the approval of the study protocol must also be provided in the manuscript.

Field-specific reporting

Please select the one below that is the best fit for your research. If you are not sure, read the appropriate sections before making your selection.

☒ Life sciences ☐ Behavioural & social sciences ☐ Ecological, evolutionary & environmental sciences

For a reference copy of the document with all sections, see nature.com/documents/nr-reporting-summary-flat.pdf

Life sciences study design

All studies must disclose on these points even when the disclosure is negative.

Sample size	Sample size was not predetermined, but based on available replicates in our high throughput platform and the minimum number of devices required to enable robust statistical comparisons between experimental conditions. For ELISA assays, at least two technical replicates were carried out for at least three experimental replicates of each platform. All sample sizes are indicated in plot captions in the manuscript.
Data exclusions	Outliers in datasets were excluded as defined by an interquartile range for PGE-2 ELISA data.
Replication	Replication is as indicated in manuscript for each experiment.
Randomization	Samples were allocated into groups in regions of a well plate and data was measured in plate-wise fashion that was independent of group location.
Blinding	Blinding was not possible or relevant to this study because samples were collected from a well plate-like platform, which was organized by investigators.

Reporting for specific materials, systems and methods

We require information from authors about some types of materials, experimental systems and methods used in many studies. Here, indicate whether each material, system or method listed is relevant to your study. If you are not sure if a list item applies to your research, read the appropriate section before selecting a response.

Materials & experimental systems

n/a	Involved in the study
☐	☒ Antibodies
☐	☒ Eukaryotic cell lines
☒	☐ Palaeontology and archaeology
☒	☐ Animals and other organisms
☒	☐ Clinical data
☒	☐ Dual use research of concern

Methods

n/a	Involved in the study
☒	☐ ChIP-seq
☒	☐ Flow cytometry
☒	☐ MRI-based neuroimaging

Antibodies

Antibodies used	Primary rabbit antibody to Vementin (Abcam, EPR3776, ab92547); Primary mouse antibody to E-Cadherin (Abcam, HECD-1, ab1416); Primary mouse antibody to CK10 (Abcam, DE-K10, ab9026); Primary rabbit antibody to CK14 (Abcam, EP1612Y, ab51054); Primary mouse antibody to ZO1 (Thermofisher, 33-9100); Primary rabbit antibody to von Willebrand Factor (Abcam, ab9378); conjucated phalloidin-iFluor 633 (Abcam, ab176758); Hoecsht 33342
Validation	Antibody validation provided by manufacturers is available from Abcam (https://www.abcam.com/) and Thermo (https://www.thermofisher.com/us/en/home.html)

Eukaryotic cell lines

Policy information about cell lines and Sex and Gender in Research

Cell line source(s)	Human gingival fibroblasts (Lifeline, FC-0095, Lot# 04920); Human dermal microvascular endothelial cells (Lonza, CC-2543); human oral keratinocytes (Lifeline, FC-0094, Lot #04971)
Authentication	Primary gingival cells were directly cultured from their source tissue (gingiva), expanded twice and cryopreserved to ensure viability, plating efficiency proper growth and morphology. Endothelial cells were cultured to be 90% pure and stained for von Willebrand factor and CD31.
Mycoplasma contamination	Lifeline cells tested negative for mycoplasma, bacterial and fungal growth for 14 days by the vendor. ATCC cells also tested negative for mycoplasma, bacteria, yeast and fungi as certified by the vendor.
Commonly misidentified lines (See ICLAC register)	All cells are primary cells and are not listed in the ICLAC register
